# Effects of Dietary Stevioside Supplementation on Feed Intake, Digestion, Ruminal Fermentation, and Blood Metabolites of Goats

**DOI:** 10.3390/ani9020032

**Published:** 2019-01-23

**Authors:** Xuefeng Han, Chaoxi Chen, Xiaoli Zhang, Yuqing Wei, Shaoxun Tang, Jiurong Wang, Zhiliang Tan, Liwei Xu

**Affiliations:** 1 Key Laboratory for Agro-Ecological Processes in Subtropical Region, National Engineering Laboratory for Pollution Control and Waste Utilization in Livestock and Poultry Production, South-Central Experimental Station of Animal Nutrition and Feed Science in Ministry of Agriculture, Hunan Provincial Engineering Research Center for Healthy Livestock and Poultry Production, Institute of Subtropical Agriculture, The Chinese Academy of Sciences (CAS), Changsha 410125, China; xfhan@isa.ac.cn (X.H.); amiy826@foxmail.com (X.Z.); wyq523990006@163.com (Y.W.); sxtang@isa.ac.cn (S.T.); jrwang@isa.ac.cn (J.W.); zltan@isa.ac.cn (Z.T.); 2 College of Life Science and Technology, Southwest University for Nationalities, Chengdu 610041, China; chaoxi8832@163.com; 3 Hunan Co-Innovation Center of Animal Production Safety (CICAPS), Changsha 410128, China

**Keywords:** sweetener, stevioside, intake, goats

## Abstract

**Simple Summary:**

This study evaluated the effects of stevioside, a natural sweetener that is an extract of Stevia, on feed intake and feeding behavior of goats. The results demonstrated that the consumption of forage was improved after supplementation with stevioside. Supplementation of stevioside resulted in improved total diet consumption of goats. These results are useful for ruminant producers to use natural sweeteners to regulate the intakes of animals.

**Abstract:**

The objective of this study was to evaluate the effects of dietary inclusion of stevioside on feed intake, feeding behavior, nutrient digestion, rumen fermentation, and serum biochemical parameters in goats. Nine male Xiangdong black goats (21.8 ± 1.5 kg of body weight) were used in a replicated 3 × 3 Latin square. All goats were fed a basal diet including concentrate and forage (chopped rice straw). The three treatments were 0, 400, or 800 mg stevioside per kg chopped rice straw on a dry matter (DM) basis. Dry matter intake of forage and total diet linearly increased (*p =* 0.03 and *p =* 0.04) with increasing stevioside in the diet. There was no effect (*p* > 0.05) of stevioside inclusion in the diets on eating time, rumination time, or total mastication time for the goats. Total volatile fatty acid (VFA) concentration in the rumen responded quadratically (*p* < 0.01), decreasing from 0 to 400 mg/kg stevioside inclusion and increasing thereafter. The inclusion of steviosid in the diets promoted a quadratic increase in the apparent total tract digestibilities of neutral detergent fiber (NDF) (*p* = 0.02) and acid detergent fiber (ADF) (*p* = 0.01). Based on the results of this experiment, it could be concluded that supplementing goat diets with stevioside at 400 mg/kg to 800 mg/kg forage (about 270 to 541 mg/kg diet) resulted in increased dry intake of forage and total diet, suggesting that stevioside has positive potential as a feed additive to improve feed intake.

## 1. Introduction

Feed intake directly affects the performance of domestic animals. Palatability of feed plays an important role in feed intake regulation, and the palatability of a feed can be defined as all the physical (appearance, texture, etc) and chemical (taste, smell, etc) characteristics of the feed that act on appetite [1]. Feed intake can be increased when some chemical components such as monosodium glutamate [2], flavor, and fragrance [3] are added to the ruminant diets to improve the taste and smell, especially when feed is unpalatable. Taste of feed is one of the important factors that can influence palatability and feed consumption by animals. Taste is associated with chemical components that can be perceived by the mouth, nose, and brain of animals. Sweetener as a taste modifier is used to affect taste of feed to enhance the palatability. Sweet flavors are associated with natural and artificial sweetening agents. Some studies have reported that natural or artificial sweetener can increase feed intake and average daily gain when they are supplemented to ruminants’ diets [4,5]. Goatcher and Church [6] observed that the sensitivity extent to sucrose among sheep, cattle, and goats is cattle > goats > sheep. Cattle prefer hay supplemented with sugar up to 100 g per kg hay compared with no sugar addition [7].

*Stevia rebaudiana* is a plant species in the genus *Stevia* of the sunflower family (Asteraceae), commonly known as candyleaf, sweetleaf, or sugarleaf. Both dried leaves of the plant as well as aqueous extracts have been used for decades as a sweetener in many countries, notably in Latin America and East Asia. The *Stevia*-derived sweeteners have sensory and functional properties superior to those of many other high-potency sweeteners and are likely to become a major source of natural sweetener for the growing food market [8].

Stevioside (13-[(2-O-beta-D-glucopyranosyl–alpha-D-glucopy-ranosyl) oxy] kaur-16-en-18-oic acid-beta-D-glucopyranosyl ester), is one of the main steviol glycosides isolated from the leaves of *Stevia rebaudiana*. Because of its stability in light, heat and low pH, high-intensity sweetness (250–300 times sweeter than sucrose), lack of caloric value, and safety [9], stevioside as a natural sweetener has broad application prospects in food and drug industry.

Studies have been conducted to evaluate the use of stevioside in the diets of non-ruminants including pigs [10,11,12] and poultry [13,14]. Those studies mostly observed that inclusion of stevioside increases the feed intake of animals. In ruminants, there was only a study involving stevioside in which the effects of a formulated additive containing 50% of protected fat, 25% of yeast culture, 5% of choline, 7% of organic zinc, 6.5% of cinnamon, and 6.5% of stevioside (as an essential oil) on rumen and rectal temperature in Hanwoo steers were investigated [15]. To our knowledge, there is no information on the use of stevioside as a sweetener in ruminant diets. Herein, we hypothesized that stevioside could increase feed intake in ruminants. Therefore, the objective of this study was to evaluate the effects of dietary stevioside inclusion in goats on feed intake and feeding behavior. Additionally, we investigated the possible effects of dietary inclusion of stevioside on rumen fermentation, digestibility, and blood serum metabolites of goats.

## 2. Materials and Methods

### 2.1. Animal Ethics

This study was carried out at the Institute of Subtropical Agriculture’s Animal Experimental Station, Changsha, China. The protocol of this experiment was approved by the Animal Care Committee (Permit No. ISA000256), Institute of Subtropical Agriculture, the Chinese Academy of Sciences, Changsha, China.

### 2.2. Design, Animals and Diet

Nine male Xiangdong Black goats with an approximate age of 12 mo and 21.8 ± 1.5 kg initial body weight (BW) were used in the study. In the production of Xiangdong Black goats, non-sire or non-breeding rams are fattened after castration. Referring to production practice of Xiangdong Black goats, all goats were castrated surgically before the trial and then allowed to recover for four weeks. Animals were penned individually in stainless metabolic cages, each cage with a concentrate and a forage trough. Cages were designed for separate collection of feces and urine. Animals had free access to fresh water throughout the entire experiment.

The animals were allotted to the three dietary treatments in a replicated 3 × 3 Latin square design with 3 periods. Each period lasted for 20 days. Based on the published results of stevioside and the test results of stevioside in goats by our group, goats were divided into three groups (S0, S400, and S800) to be, respectively, fed a diet with 0, 400, and 800 mg stevioside per kg forage on a dry matter (DM) basis. Goats were fed a diet containing concentrate and forage twice daily. The ingredients and chemical composition of the basal diet are shown in Table 1. 

The concentrate was given to each animal twice daily at 0830 and 1830 h. The amounts of concentrate for individual goats per feeding were 0.5% of their BW. Body weight was determined at the beginning and the end of each experimental period before feeding. The amounts of concentrate for individual goats were adjusted according to their BW at the beginning of each period. This concentrate at each feeding was always completely eaten by each goat during the entire experiment. The forage was rice straw chopped into approximately 5-cm pieces. The chopped rice straw was provided to goats twice (0800 and 1800 h) per day. The stevioside, which is the white powder extracted from *Stevia rebaudiana* leaves, was provided by Runde Biotechnology CO., LTD. (Qingdao, China). Samples of stevioside were analyzed in the beginning of the trial to determine the purity (97%) by high performance liquid chromatography (HPLC). Stevioside was dissolved in 125 mL of water and the water solution was then thoroughly mixed with 250 g chopped rice straw by hand before each feeding of rice straw. Chopped rice straws were fed for ad libitum intake to permit at least 10% refusals, and forage refusals from each goat were removed before the next feeding of chopped rice straw.

### 2.3. Sampling, Data Collection, and Chemical Analyses

Adaptation to experimental diets took place from days 1 to 11, total collection of feces from days 12 to 17, mastication observation on day 18, blood sampling on day 19, and ruminal sampling on days 19 and 20.

Chopped rice straw and concentrates were sampled for each period to determine DM concentration. Amounts of feed offered and refused by an individual animal at each feeding were recorded throughout the whole period. The forage refusals of each animal per feeding were sampled immediately after they were collected and weighed then dried at 65 °C for 48 h. The dried samples of forage refusals were stored for generating composite samples per animal in each period. Then, these composite samples were ground to pass a 1-mm sieve with a mill (FW-100, Yongguangming Ltd., Beijing, China) for subsequent DM analysis. Contents of DM of the samples were used to calculate daily intake of individual goats.

From days 12 to 17, feces from all goats were collected daily in stainless steel containers with stainless steel gauze to determine total tract apparent digestibility of dietary nutrients. Total fecal output was mixed and weighed daily. A representative sample (10%) was taken and stored at −20 °C. The fecal samples were subsequently thawed, dried in an oven at 65 °C, and composited per animal and experimental period, and then ground through a 1-mm screen with a mill (FW-100, Yongguangming Ltd., Beijing, China) for chemical composition analyses. Apparent total tract digestibility was calculated by subtracting fecal output from intake, divided by intake of nutrients.

On day 18, after the six-day feces sample collection, feeding behavior were recorded over a 24 h period [16,17]. The feeding behavior was evaluated by visual inspection of each animal performed by six trained examiners divided in pairs, who took turns at each 3 h shift. Nighttime observations were conducted using artificial lighting. The recorded activities were eating, ruminating, and resting. Time devoted to eating or rumination by the each animal is estimated by adding all “eating minutes” or “ruminating minutes”. Total time spent mastication was calculated as the sum of total time spent eating and ruminating.

On day 19, blood were collected from the jugular vein at 0, 2.5, and 6 h after the morning feeding by trained personnel using a vacuum serum tube (5 mL, Aosaite medical instrument Co., LTD, Heze, China). Blood without anticoagulant was allowed to clot for 1 h at room temperature and was then centrifuged at 3000× *g* for 10 min at 4 °C. All serum samples were free of hemolysis and were frozen at −20 °C until analyzed for serum metabolites by Mindray BS-230 Chemistry Analyzer (Shenzhen, China). The blood biochemical indexes included glucose (GLU), total protein (TP), albumin (ALB), globulin (GLB), triglyceride (TG), and total cholesterol (TC).

On days 19 and 20, rumen fluid samples were collected at 0, 2.5, and 6 h after morning feeding via the mouth of goat using a plastic pipe and approximately 10 mL of ruminal fluid was extracted and discarded [18,19]. The first 10-mL sample was discarded to eliminate saliva contamination that would falsely elevate the pH level. Then, at least 50 mL of ruminal fluid was collected and placed into 50-mL propylene conical tubes (Thermo Scientific Inc., Waltham, MA, USA). The rumen fluid was first strained through four layers of cheesecloth and then used for analyses of pH and volatile fatty acid (VFA) concentrations. The pH of filtered ruminal fluid was measured immediately after sampling by using a pH meter (FE20 FiveEasy Benchtop pH Meter, Mettler-Toledo, LLC, OH, USA), which was calibrated before each sampling time using standard buffers (pH 4.0 and 7.0). Samples of rumen fluid were centrifuged for 15 min at 10,000× *g* at 4 °C. Aliquots (1.0 mL) of the supernatants were acidified with 25% metaphosphoric acid (1:4 vol/vol; acid:rumen fluid) [20]. The mixtures were vigorously hand shaken and stored at −20 °C for subsequent VFA analysis. The remaining supernatants were frozen at −20 °C for later determination of NH_3_-N concentrations.

Samples of chopped rice straw and concentrates were ground with a mill (FW-100, Yongguangming Ltd., Beijing, China) using a 1-mm sieve and sealed properly until needed for a laboratory analysis to determine the chemical composition according to the Association of Official Analytical Chemits [21]. Dry matter (DM) content of was determined by drying samples for 24 h at 105 °C in a forced-air oven. Samples were ashed at 550 °C for 12 h in a muffle furnace, and organic matter (OM) content was determined as the difference between 100 and the percentage of ash. Ether extract (EE) were analyzed in a Soxhlet extractor (SOX416 Soxtherm, C. Gerhardt GmbH & Co. KG, Königswinter, Germany) according to the instrumental manual. Crude protein (CP = N × 6.25) was determined by a Kjeldahl analysis. The concentrations of neutral detergent fiber (NDF) and acid detergent fiber (ADF) were sequentially determined using a Fibretherm FT 12 Fiber Analyzer (C. Gerhardt GmbH & Co. KG, Königswinter, Germany) according to the methodology supplied by the company, which is based on the methods described by Van Soest et al. [22].

Concentration of NH_3_-N in ruminal fluid was determined according to the procedure of Weatherburn [23]. The frozen samples of acidified rumen fluid were thawed and centrifuged at 15,000× *g* for 10 min at 4 °C before the VFA profiles were measured by gas chromatography (GC7890A, Agilent, Santa Clara, CA, USA), according to the method described by Wang et al. [24].

### 2.4. Statistical Analysis

Data were analyzed using PROC MIXED (SAS Institute, Cary, NC, USA) [25], according to the model:

Yijkl = μ + Si + Pj + Ck(i) + Tl + STil + εijkl,(1)

where Yijkl is the response variable, μ is the overall mean, Si is the effect of square i, Pj is the effect of period j, Ck(i) is the random effect of goat k (within square i), Tl is the effect of treatment l, STil is the interaction between square i and treatment l, and εijkl is the residual error. Sampling times were added to the model for the analysis of ruminal fermentation characteristics and plasma metabolites, and were analyzed as repeated measures by using PROC MIXED. Orthogonal polynomial contrasts were used to test linear and quadratic effects of stevioside concentration in the diet. Results were reported as least squares means ± standard error of the means. Significance was declared at *p* < 0.05.

## 3. Results

### 3.1. Effects of Stevioside on Feed Intake and Mastication Activities

The inclusion of stevioside in the diets promoted a linear increase in the dry matter intakes of forage ((*p* = 0.03) and diet (*p* = 0.04; Table 2). The eating time, rumination time, and total mastication time were not affected (*p* > 0.05) by the addition of stevioside to the diets of the goat.

### 3.2. Effects of Stevioside on Rumen Fermentation

The results showed that pH increased quadratically (*p* = 0.02) to increasing stevioside inclusion in the diet (Table 3). NH_3_-N was not different among groups (*p* > 0.05). However, total VFA concentration quadratically decreased (*p* < 0.01) as stevioside concentration increased. The proportions of acetate, propionate, butyrate, and valerate did not differ among stevioside levels (*p* > 0.05). As a result, the acetate:propionate ratio was not affected (*p* > 0.05) by the addition of stevioside. There was a quadratic response of stevioside on proportions of isobutyrate (*p* < 0.01) and isovalerate (*p* = 0.02).

### 3.3. Effects of Stevioside on Apparent Total Tract Digestibility

Apparent total tract digestibility of DM, OM, and CP were not affected (*p* > 0.05) by the tested levels of stevioside (Table 4). However, apparent digestibility of NDF and ADF quadratically increased (*p* = 0.02 and *p* = 0.01), where NDF and ADF digestibilities increased from 0 to 400 mg/kg stevioside and then decreased from 400 to 800 mg/kg stevioside.

### 3.4. Effects of Stevioside on Serum Parameters

There were no differences (*p* > 0.05) in the concentrations of GLU, TP, ALB, GLB, TG, and TC among treatments (Table 5). The serum concentrations of GLU, TP, ALB, GLB, TG, and TC were not affected (*p* > 0.05) by the addition of stevioside to the diets of the goats (Table 5).

## 4. Discussion

The current study evaluated the effects of stevioside supplementation on intake, apparent digestibility, rumen fermentation, and blood metabolite parameters. Although stevioside treatments did not affect mastication behavior, they increased total dry matter and forage intakes of goats. We speculated that stevioside could increase the palatability of rice straw by increasing the sweetness, and then promote goat eating more rice straw, which ultimately resulted in increased dietary intake. Cho et al. [15] reported that a feed additive containing 6.5% stevioside, which was used as an essential oil there, supplemented at a rate of 0.1% of diet, did not influence feed intake of steers. According to the additive formulation and steer feed intakes in the study of Cho et al. [15], the supplementation amount of stevioside was calculated as about 65 mg/kg diet; however, the supplementation concentrations of stevioside are about 270 and 541 mg/kg diet in the current study. Because stevioside was only one component of the steer feed additive [15] and its adding concentration was also far below those in our study, there is little comparability between this study and that of Cho et al. [15] in terms of the effects of stevioside on feed intake.

The current study showed that dietary stevioside had an impact on ruminal pH, total VFA concentration, and isobutyrate and isovalerate proportions. Total VFA concentration responded quadratically, decreasing from 0 to 400 mg/kg stevioside inclusion and increasing thereafter. Corresponding to changes in total VFA concentration, ruminal pH responded quadratically, increasing from 0 to 400 mg/kg stevioside and then decreasing. Though the differences in ruminal pH between groups were statistically significant, the change in ruminal pH value between treatments was small (only 0.08) and the pH values of these three groups were within the normal range, which implies rumen fermentation function is normal. The inclusion of stevioside caused a quadratic response in total VFA concentration, and the proportions of isobutyrate and isovalerate, however had no effect on the proportions of acetate, propionate, butyrate and valerate, and acetate:propionate ratio. Although the proportions of predominant VFA forms (acetate, propionate, and butyrate) did not differ across treatments, total VFA concentration responded to dietary stevioside inclusion, which possibly indicates stevioside had an effect, for example by affecting the yield of acid, on rumen fermentation in goats. Besides a sweetening property, there are several physiological effects of stevia and its extracts, including antimicrobial property [26,27]. Stevioside has also been reported to have antibacterial activity [28], which implies stevioside might influence the microbial population of the gastrointestinal tract of animals. However, under the supplementation levels of about 270 and 541 mg/kg diet in the current study, whether stevioside could affect ruminal fermentation by influencing rumen micro-organisms is unclear. Further in vivo and in vitro studies are needed to explore the effects of stevioside on gastrointestinal microbes and fermentation in ruminants.

In the present study, total tract apparent digestibilities of NDF and ADF were increased quadratically with the inclusion of stevioside. The inclusion of stevioside in the diets of goats caused a significant increase in forage and diet intakes, which means that the intakes of NDF and ADF would also increase accordingly. As forage intake increases, feed passage rate through the gastrointestinal tract would be faster and fiber digestibility usually decreases [29,30,31]. However, our results showed that the amount of forage and diet increased with increasing digestibilities of NDF and ADF. The reason for this is unclear.

In the present study, the serum levels of glucose, total protein, albumin, globulin, triglyceride, and total cholesterol were not affected by the inclusion of stevioside in the diets, and this demonstrated stevioside added at levels up to 541 mg/kg diet did not influence the energy and nutritional status of goats.

## 5. Conclusions

The results of the present study suggest that the addition of increasing amounts of stevioside to the diet of goats increased DMI, and NDF and ADF digestibility. This study showed that dietary stevioside supplementation may promote higher feed intake of goats, which indicate a potential application of stevioside as a bio-sweetener in ruminant diets. Further studies are needed to investigate the influences of diet supplementation with stevioside on the performance of goats.

## Figures and Tables

**Table 1:** Ingredients and chemical composition of diet in the experimental *.

Items	Diet *	
	Concentrate	Forage
Ingredient, % of DM		
Corn	51.2	
Wheat bran	19.6	
Soybean meal	15.1	
Urea	2.01	
Calcium carbonate	0.42	
Vegetable oil	5.16	
Salt	1.51	
Mineral and vitamin mix	5.02	
Rice straw		100.0
Chemical composition, % of DM		
Digestible energy (Mcal/kg)	3.61	1.23
CP	22.4	5.60
EE	2.78	0.80
Ash	10.8	10.8
NDF	17.7	65.7
ADF	9.94	48.5

* Diet included both concentrate and forage; Mineral and vitamin mix contained 8.5 g/kg Mg, 4 g/kg Zn, 510 mg/kg Fe, 750 mg/kg Cu, 5.1 g/kg Mn, 11 mg/kg Co, 30 mg/kg I, 5 mg/kg Se, 104,400 IU/kg vitamin A, 17,500 IU/kg vitamin D, and 2,200 IU/kg vitamin E; DM: Dry matter; CP: crude protein; EE: ether extract; Ash: ash in animal feed; NDF: neutral detergent fiber; ADF: acid detergent fiber.

**Table 2:** Effects of stevioside on feed intake and mastication of goats *.

Items	Stevioside, mg/kg Forage DM			SEM	*p* Value	
	0	400	800		Linear	Quadratic
Dry mater intake (DMI), g/d						
Forage	369.9	387.4	393.4	6.63	0.03	0.49
Concentrate	188.4	187.1	188.3	0.91	0.93	0.30
Total	558.3	574.0	581.7	7.20	0.04	0.67
Mastication, min/d						
Eating	422.2	430.6	428.9	20.99	0.83	0.85
Rumination	555.2	572.0	577.6	23.80	0.53	0.85
Total mastication	977.4	1002.6	1006.5	28.12	0.49	0.76

* Stevioside were added to the forage-rice straw at 0, 400 and 800 mg/kg DM, respectively; DMI: Dry mater intake; SEM = standard error of the mean; differences were considered significant if *p* ≤ 0.05.

**Table 3:** Effects of stevioside on rumen fermentation in goats *.

Items	Stevioside, mg/kg Forage DM			SEM	*p* Value	
	0	400	800		Linear	Quadratic
pH	6.58	6.66	6.62	0.02	0.16	0.02
NH_3_-N, mg/dL	9.40	9.04	8.99	0.59	0.63	0.84
Total VFA, μmol/mL	73.7	64.8	70.8	2.05	0.32	<0.01
VFA, mol/100 mol						
Acetate	77.0	76.3	76.3	0.29	0.09	0.45
Propionate	15.1	15.4	15.7	0.25	0.10	0.99
Butyrate	5.92	5.89	6.10	0.17	0.45	0.55
Isobutyrate	0.78	0.97	0.81	0.03	0.53	<0.0001
Valerate	0.47	0.51	0.49	0.02	0.48	0.21
Isovalerate	0.79	0.86	0.69	0.04	0.05	0.02
A/P ratio	5.15	5.07	4.93	0.09	0.08	0.78

* Stevioside were added to the forage-rice straw at 0, 400 and 800 mg/kg DM, respectively; VFA: volatile fatty acid; A/P ratio: Acetate/propionate ratio; SEM: standard error of the mean; Differences were considered significant if *p* ≤ 0.05.

**Table 4:** Effects of stevioside on apparent total tract digestibility in goats *.

Items	Stevioside, mg/kg Forage DM			SEM	*p* Value	
	0	400	800		Linear	Quadratic
DM, %	59.3	61.3	58.9	0.87	0.77	0.08
OM, %	62.8	64.9	62.7	0.91	0.95	0.11
CP, %	64.9	64.4	64.0	0.76	0.45	0.95
NDF, %	55.0	62.2	60.3	1.14	0.01	0.02
ADF, %	67.2	75.3	73.8	1.59	0.01	0.01

* Stevioside were added to the forage-rice straw at 0, 400 and 800 mg/kg DM, respectively; SEM: standard error of the mean; differences were considered significant if *p* ≤ 0.05; DM: dry matter; OM: organic matter; CP: Crude protein; NDF: neutral detergent fiber; ADF: acid detergent fiber.

**Table 5:** Effects of stevioside on serum metabolites in goats *.

Items	Stevioside, mg/kg Forage DM			SEM	*p* Value	
	0	400	800		Linear	Quadratic
GLU, mmol/L	0.60	0.60	0.61	0.01	0.22	0.59
TP, g/L	63.9	65.4	64.8	0.75	0.42	0.24
ALB, g/L	29.1	29.5	29.5	0.27	0.22	0.50
GLB, g/L	34.7	36.2	35.5	0.56	0.37	0.12
TG, mmol/L	0.43	0.43	0.45	0.02	0.41	0.72
TC, g/L	4.22	4.14	4.09	0.05	0.09	0.80

* Stevioside were added to the forage-rice straw at 0, 400 and 800 mg/kg DM, respectively; GLU: glucose; TP: total protein; ALB: albumin; GLB: globulin; TG: triglyceride; TC: total cholesterol; SEM: standard error of the mean; differences were considered significant if *p* ≤ 0.05.
